# Psychiatric comorbidities and concurrent substance use among people who inject drugs: a single-centre hospital-based study

**DOI:** 10.1038/s41598-023-45633-y

**Published:** 2023-11-04

**Authors:** Hadiya Kar, Abdul Majid Gania, Altaf Bandy, Nizam ud din Dar, Farhana Rafiq

**Affiliations:** 1 Department of Psychiatry, SKIMS Medical College, Bemina, Srinagar, 190018 India; 2 College of Medicine, Shaqra University, Shaqra, 15571 Kingdom of Saudi Arabia

**Keywords:** Diseases, Medical research, Risk factors

## Abstract

The management of people who inject drugs (PWID) is compounded by the presence of psychiatric comorbidities leading to frequent relapses and poor treatment outcomes. Early identification and treatment of psychiatric comorbidities should be included in the management to enhance treatment outcomes. The objective of this study was to estimate the prevalence of psychiatric comorbidities and concurrent substance use among opioid injectors. This hospital-based, cross-sectional study was conducted from March 2021 to August 2022. This study included opioid injectors of all ages and both sexes. The Mini International Neuropsychiatric Interview-7 *(MINI-7*) and WHO-ASSIST were used to determine psychiatric comorbidities and concurrent substance use, respectively. Both crude and adjusted odds ratios were calculated to assess associations among demographic variables, concurrent substance use and psychiatric comorbidities. Among the 328 opioid injectors, the overall prevalence of psychiatric comorbidities was 88.1%, with the majority (68.6%) having more than one comorbidity. The most common psychiatric comorbidities were panic disorder (41.2%), social anxiety disorder (40.5%), and antisocial personality disorder (39.3%). Concurrent use of alcoholic beverages doubled the risk of ASPD (odds ratio 2.14 (1.24–3.72)). Cocaine (odds ratio 2.36 (1.10–5.03)) and amphetamines (odds ratio 7.68 (2.21–26.65)) increased the risk of OCD. Daily heroin injections were negatively associated (odds ratio 0.18 (0.03–0.94)) with psychotic disorders. Younger age (adjusted odds ratio 0.20 (0.79–0.53)) and never married status (adjusted odds ratio 2.62 (1.06–6.47)) were the only significant variables in the regression analysis. In conclusion, opioid injectors had a higher prevalence of numerous psychiatric comorbidities. The most common comorbidity was anxiety disorders. Concurrent use of tobacco, cannabis, cocaine, inhalants, etc., greatly increased the risk of psychiatric comorbidities.

## Introduction

Worldwide, 11 million individuals use injection drugs^1^. Approximately 8.5 million people in India alone inject drugs. Except for the Union Territories of Andaman and Nicobar and Lakshadweep, injection drug use (IDU) has been documented in all states across the nation. In Jammu and Kashmir, there are an estimated 25,000 people who inject drugs (PWID)^2^. Injection drug use is the most dangerous method of drug abuse. Following an injection, a large bolus of the drug is instantly carried into the bloodstream and rapidly delivered to the brain. As a consequence, PWID are more likely to experience dependence and overdose than people who abuse drugs by other routes. Maladaptive thoughts in PWID with negative affect undermine the desire to care for themselves. This attitude poses a risk for the transmission of blood-borne diseases^3^. Worldwide, previous research has shown that heroin is the most abused substance (53%), followed by methamphetamine (43%) and cocaine (40%)^4^. In Asia, heroin is the most commonly injected substance, and approximately 70–80% of PWID have a background of polydrug use^5^. In India, PWID opt to inject one or more opioid drugs^2^.

Comorbid psychiatric illnesses complicate the treatment of such patients, adding to the burden experienced by PWID. The term ‘comorbidity’ was introduced in medicine to refer to cases with a distinct clinical course in addition to the index illness. The term ‘dual diagnosis’ (DD) refers to the coexistence of a specific substance use disorder (SUD) and other psychiatric illnesses (non-SUD)^6^. Psychiatric comorbidities can be independent of either drug use or substance use. These illnesses can contribute to substance use disorders via a combination of biological and environmental pathways^7^. According to previous studies, opioid users have the highest prevalence of dual diagnoses^8^. Approximately 40% of PWID have psychiatric comorbidities, and 51% of psychiatric patients have substance use disorders^5,9^. Polydrug abuse, which is prevalent among PWID, is ascribed to the presence of comorbid psychiatric disorders^10^. Patients with substance abuse and psychiatric comorbidities are difficult to manage due to a variety of factors. Multiple clinical presentations, the severity of symptoms, a greater number of relapses, a poor prognosis, increased hospitalization length of stay, and higher suicide rates result in lower chances of recovery^7,11^. The similarities in the presentation of psychiatric comorbidities with substance intoxication and withdrawal effects make proper and timely diagnosis challenging^12^. According to previous studies, clinicians frequently underdiagnose psychiatric comorbidities among drug users, which has an impact on treatment plans for such patients^13^. As a result, greater vigilance towards comorbidities is needed when managing patients with substance use disorders for holistic patient management^6^.

For decades, the Kashmir region has been in turmoil^14^. Several studies have found an increase in the prevalence of psychiatric illnesses such as posttraumatic stress disorder (PTSD) and depression^15^. Due to political turmoil, the state has experienced extended lockdown periods in recent years, during which residents have been confined to their homes. Conflict, traumatic experiences, economic development constraints, and the breakdown of conventional social support mechanisms have all contributed to psychological distress in this population^16–18^. Young people represent the most susceptible section of society for a variety of reasons, including uncertainty about the future, a lack of employment, an internet ban, overwhelming fear, and a lack of self-esteem^14^. This has paved the way for a rise in psychiatric disorders and poor coping strategies such as drug abuse among young people. Substance abuse has largely gone undetected^19^. Various sociocultural and geographical factors play an essential role in the onset, maintenance, and treatment of substance use disorders^20^. According to recent research on substance use in Jammu and Kashmir, the prevalence of substance use is 1.95%^21^, with a total number of 25,000 PWID in the entire state^2,21^. While the relationship between various drugs and psychiatric comorbidities has been studied in various parts of the world, few studies on injection drug abuse and comorbid psychiatric disorders in Kashmir have been conducted^5,7,22–24^.

In the above context, this study was carried out to estimate the prevalence of comorbid psychiatric disorders among opioid-injecting drug users. Furthermore, this study also assessed the prevalence of concurrent substance use and its association with psychiatric disorders.

## Results

Of the 328 opioid injectors enrolled in this study, 324 (98.8%) and 4 (1.2%) were males and females, respectively. The age of the participants ranged from 17 to 42 years, with a mean of 25.31 ± 5.32 years. The majority, 188 (57.3%), of the study participants were in the 15–24 age group. A total of 121 (36.9%) participants had an intermediate level of education, and 74 (22.6%) had a high school certificate. Only 97 (29.6%) of the participants were currently married.

The study participants had initiated opioid injections at a mean age of 21.77 ± 5.65 years. Peer pressure (47.9%) was the most common reason for the initiation of injections, whereas 14.6% and 11.3% reported initiation due to curiosity and pleasure, respectively. Most (64.3%) of the participants were referred for treatment by friends, followed by family (20.7%) and physicians (9.8%). Of the opioid users, only 36.3% reported a family history of substance use. Psychiatric comorbidities were present in 88.1%, of which the majority (68.6%) had more than one psychiatric comorbidity (Table 1).

**Table 1:** Baseline characteristics of opioid injectors (n = 328).

Characteristic	Frequency (n)	Percentage (%)
Sex		
Male	324	98.8
Female	4	1.2
Age in years	25.31 ± 5.32 (range: 17–42)	
Age categories		
15–24 years	188	57.3
25–34 years	107	32.6
35–44 years	33	10.1
Educational status		
Illiterate	3	0.9
Literate without formal education	9	2.7
Primary school certificate	24	7.3
Middle school certificate	51	15.5
Matriculation/high school certificate	74	22.6
Intermediate	121	36.9
Diploma not equal to degree	4	1.2
Graduate and above	40	12.2
Professional or honours	2	0.6
Marital status		
Never married	216	65.9
Currently married	97	29.6
Separated	4	1.2
Others	11	3.4
Income generation in last 12 months		
Yes	262	79.9
No	66	20.1
Occupation in the last 12 months		
Professional	6	1.8
Semiprofessional	8	2.4
Clerical	2	0.6
Shop owner	62	18.9
Farmer	6	1.8
Skilled worker	111	33.8
Semiskilled worker	42	12.8
Unskilled worker	39	11.9
Unemployed	52	15.9
Income		
≤ 10,000	61	18.6
11–20k	92	28.0
21–30k	45	13.7
31–40k	36	11.0
41–50k	32	9.8
> 50k	62	18.9
Residence		
Yes	319	97.3
No	9	2.7
Current living arrangement		
Joint family	59	18.0
Nuclear family	262	79.9
With friends	7	2.1
Age of initiation of injecting behaviour	21.77 ± 5.65 (range = 10–38)	
Referral paths		
Self	17	5.2
Family	68	20.7
Friends	211	64.3
Physicians	32	9.8
Reasons for injecting opioids		
Peer pressure	157	47.9
Stress	32	9.8
Curiosity	48	14.6
Pleasure/fun	37	11.3
Prescription	3	0.9
Multiple reasons	51	15.5
Family history of substance use		
No	209	63.7
Yes	119	36.3
Psychiatric disorders		
Absent	39	11.9
One comorbidity	64	19.5
Multiple comorbidities	225	68.6

The most common psychiatric comorbidity was panic disorder (41.2%), followed by social anxiety disorder (40.5%) and antisocial personality disorder (39.3%). Major depressive disorder (17.7%), alcohol use disorder (14.6%), bipolar affective disorder (8.23%), obsessive–compulsive disorder (11%), and psychotic disorders (3%) were the other frequent disorders found in the study. Most of the study participants (70.4%) had low intent for suicidality. Medium and high intent was found in 20.4% and 9.1%, respectively (Table 2).

**Table 2:** Prevalence of psychiatric disorders in the study population (n = 328).

Psychiatric disorders	Frequency (n)	Percentage (%)
Major depressive disorder	58	17.7
Bipolar and related disorders	27	8.23
Panic disorder	135	41.2
Agoraphobia	35	10.7
Social anxiety disorder	133	40.5
Obsessive–compulsive disorder	36	11.0
Posttraumatic stress disorder	28	8.5
Alcohol use disorder	48	14.6
Substance use disorder (nonalcohol)	108	32.9
Psychotic disorders and mood disorders with psychotic features	10	3.0
Anorexia nervosa	0	0
Bulimia nervosa	4	1.2
Binge eating disorder	14	4.3
Generalized anxiety disorder	72	22.0
Antisocial personality disorder	129	39.3
Suicidality		
Low intent	231	70.4
Medium intent	67	20.4
High intent	30	9.1

Codeine, oral tramadol, tapentadol, and the use of multiple drugs over time by opioid injectors showed a significant (*P* < 0.05) relationship with the development of psychiatric comorbidities. Using a combination of drugs was negatively associated with psychiatric comorbidities in people who inject drugs (Table 3).

**Table 3:** Relationship between other opioids used by opioid injectors and comorbid psychiatric conditions (n = 328).

Opioids	Frequency	Comorbidities	*P* value	Odds ratio with 95% CI
Opium				
Yes	7	7	0.60^$^	NA
No	321	282		
Morphine				
Yes	2	2	1.0^$^	NA
No	326	287		
Codeine				
Yes	194	182	0.00*	3.82 (1.86–7.86)
No	134	107		
Oral tramadol				
Yes	175	168	0.00*	6.34 (2.71–14.85)
No	153	121		
Tapentadol				
Yes	96	90	0.04*	2.48 (1.01–6.14)
No	232	199		
Combination				
Yes	55	41	0.00*	0.29 (0.14–0.61)
No	273	248		
Multiple drugs over time				
Yes	166	162	0.00*	11.16 (3.86–32.22)
No	162	127		

^$^Fisher’s exact test, *Significant.

Further analysis showed an association of concurrent drug use with some major psychiatric disorders. Alcoholic beverage intake doubled the risk (odds ratio 2.14 (1.24–3.72) of ASPD, while cannabis abuse nearly tripled (odds ratio 2.95 (1.28–6.80) the risk of MDD. Cannabis also increased (odds ratio 1.93 (1.13–3.30)) the risk of ASPD. Cocaine (odds ratio 2.36 (1.10–5.03) and amphetamine (odds ratio 7.68 (2.21–26.65) increased the risk of OCD in opioid injectors. Inhalants increased the risk of ASPD by 2.5 (2.59 (1.55–4.33)) and OCD by 2.4 (odds ratio 2.44 (1.19–5.01)) (Table 4).

**Table 4:** Bivariate analysis of the association of concurrent substance use and daily heroin use with ASPD, MDD and OCD among opioid injection users (n = 328).

Substance	MDD		*P* value	Odds ratio with 95% CI	ASPD		*P* value	Odds ratio with 95% CI	OCD		*P* value	Odds ratio with 95% CI
	P	A			P	A			P	A		
Tobacco												
Yes	58	261	0.22^$^	NA	129	190	0.13^$^	NA	36	283	8.17^$^	NA
No	0	9			0	9			9	0		
Alcoholic beverages and others												
Yes	44	201	0.82	1.07 (0.61–1.84)	107	138	0.00*	2.14 (1.24–3.72)	27	218	0.86	1.01 (0.45–2.26)
No	14	69			22	61			9	74		
Cannabis												
Yes	51	192	0.00*	2.95 (1.28–6.80)	105	138	0.01*	1.93 (1.13–3.30)	29	214	0.34	1.51 (0.63–3.58)
No	7	78			24	61			7	78		
Cocaine												
Yes	12	51	0.75	1.12 (0.55–2.26)	23	40	0.61	0.86 (0.48–1.52)	12	51	0.02*	2.36 (1.10–5.03)
No	46	219			106	159			24	241		
Amphetamines												
Yes	1	10	0.69^$^	0.45 (0.05–3.63)	5	6	0.75^$^	1.29 (0.38–4.34)	5	6	0.00*	7.68 (2.21–26.65)
No	57	260			124	193			31	286		
Inhalants												
Yes	16	65	0.57	1.20 (0.63–2.27)	46	35	0.00*	2.59 (1.55–4.33)	15	66	0.01*	2.44 (1.19–5.01)
No	42	205			83	164			21	226		
Sedatives												
Yes	29	138	0.88	0.95 (0.54–1.68)	72	95	0.15	1.38 (0.88–2.15)	22	145	0.19	1.59 (0.78–3.23)
No	29	132			57	104			14	147		
Hallucinogens												
Yes	13	35	0.06	1.93 (0.95–3.95)	19	29	1.0	1.01 (0.54–1.89)	7	41	0.38	1.47 (0.60–3.59)
No	45	235			110	170			29	251		
Heroin intake												
Daily intake	56	256	0.74^$^	1.53 (0.33–6.92)	120	192	0.15	0.48 (0.17–1.33)	33	279	0.39^$^	0.51 (0.13–1.89)
Other than daily	2	14			9	7			3	13		

*P* present, *A* absent, *MDD* major depressive disorder, *ASPD* anti-social personality disorder, *OCD* obsessive compulsive disorder.

^$^Fisher’s exact test; *Significant.

The risk of developing anxiety as a psychiatric comorbidity was increased by the use of tobacco (odds ratio 7.33 (1.49–35.95), alcoholic beverages (odds ratio 2.02 (1.21–3.38), cannabis (odds ratio 2.02 (1.21–3.38), cocaine (odds ratio 2.81 (1.40–5.65), inhalants (odds ratio 2.07 (1.15–3.72), sedatives (odds ratio 2.30 (1.43–3.69), and hallucinogens (odds ratio 4.13 (1.70–10.06). Daily heroin intake was negatively associated with psychotic disorders (odds ratio 0.18 (0.03–0.94)) (Table 5).

**Table 5:** Bivariate analysis of the association of concurrent substance use and daily heroin use with psychiatric and anxiety disorders among opioid injection users (n = 328).

Substance	Psychotic disorders		*P* value	Odds ratio with 95% CI	Anxiety disorders		*P* value	Odds ratio with 95% CI
	P	A			P	A		
Tobacco								
Yes	10	309	1.0^$^	NA	216	103	0.00^$^	7.33 (1.49–35.95)
No	0	9			2	7		
Alcoholic beverages and others								
Yes	8	237	1.0^$^	1.36 (0.28–6.57)	173	72	0.00*	2.02 (1.21–3.38)
No	2	81			45	38		
Cannabis								
Yes	10	233	0.06^$^	NA	170	73	0.00*	2.02 (1.21–3.38)
No	0	85			48	37		
Cocaine								
Yes	0	63	0.21^$^	NA	52	11	0.00*	2.81 (1.40–5.65)
No	10	255			166	99		
Amphetamines								
Yes	0	11	1.0^$^	NA	9	2	0.34	2.32 (0.49–10.95)
No	10	307			209	108		
Inhalants								
Yes	0	81	0.12^$^	NA	63	18	0.01*	2.07 (1.15–3.72)
No	10	237			155	92		
Sedatives								
Yes	2	165	0.05^$^	0.23 (0.04–1.10)	126	41	0.00*	2.30 (1.43–3.69)
No	8	153			92	69		
Hallucinogens								
Yes	3	45	0.16^$^	NA	42	6	0.00*	4.13 (1.70–10.06)
No	7	273			176	104		
Heroin intake								
Yes	8	304	0.04^$^	0.18 (0.03–0.94)	204	108	0.11^$^	0.26 (0.06–1.20)
No	2	14			14	2		

*P* present, *A* absent.

^$^Fisher’s exact test; *Significant.

In the binary logistic regression, younger age and never married status were significantly associated with the development of psychiatric comorbidities. Younger age (adjusted odds ratio 0.20 (0.79–0.53)) was a protective factor, while never married status ((adjusted odds ratio 2.62 (1.06–6.47)) increased the odds of psychiatric comorbidities (Table 6).

**Table 6:** Logistic regression analysis of the association of demographics and concurrent drug use with the presence of psychiatric comorbidities.

Binary logistic regression									
Variables included in the analysis		B	S.E.	Wald	df	Sig.	Exp (B)	95% CI for Exp (B)	
								Lower	Upper
Step 1	Age (1)	− 1.582	0.487	10.551	1	0.001	0.205	0.079	0.534
	Education (1)	0.138	0.427	0.105	1	0.746	1.148	0.497	2.652
	Marital status (1)	0.965	0.461	4.381	1	0.036	2.625	1.063	6.479
	Income (1)	− 0.493	0.420	1.381	1	0.240	0.611	0.268	1.390
	Current living arrangements (1)	0.559	0.450	1.547	1	0.214	1.749	0.725	4.222
	Alcoholic beverages (beer, wine, spirits) (1)	− 0.219	0.450	0.238	1	0.626	0.803	0.332	1.940
	Cannabis (1)	− 0.644	0.441	2.130	1	0.144	0.525	0.221	1.247
	Cocaine (1)	− 0.568	0.803	0.501	1	0.479	0.567	0.117	2.734
	Inhalants (1)	− 0.915	0.659	1.925	1	0.165	0.401	0.110	1.459
	Sedatives (1)	− 0.992	0.430	5.316	1	0.021	0.371	0.160	0.862
	Constant	4.511	1.100	16.806	1	0.000	91.026		

## Discussion

Comorbid psychiatric illnesses with injection drug use complicate management and put both caregivers and affected individuals in difficult situations. Understanding the issue of psychiatric comorbidities and concurrent substance use among opioid people who inject drugs is important in the context of pharmacotherapy and improved treatment outcomes.

The majority of our participants were male. Most of the participants were in the 15–24 age group, with a mean age of 25 ± 5.32 years. More than half of our participants had never been married, and the majority lived with nuclear family. The sociodemographic profiles of our participants were similar to those of participants in previous studies conducted by Yasir et al.^21^, Andreecia et al.^25^, and Trouiller P et al.^7^. Similar findings were reported by Nizam Ud Din et al. in their study on substance users^26^. However, in the study conducted by Andreecia et al.^25^, the majority of the participants were married and older. Our study also included participants < 18 years of age, which could partly explain the lower age distribution and never married status of most participants. Equal representation of subjects from nuclear and joint families was reported by Yasir et al.^21^, while in our study, 79.9% had nuclear families. This can be explained by the difference in the settings (community vs. hospital-based) in which the studies were conducted.

In this study, all the participants were injecting heroin. Yasir Hassan et al.^21^ and Trouiller P et al.^7^ also reported 91.12% and 100% heroin users among PWID, respectively. However, Armstrong et al.^27^ found buprenorphine to be the major injectable in 77% and heroin in 18% of the participants. This might be related to the availability of heroin in the study area. Nearly all of the participants had used tobacco products in the last three months. Alcoholic beverages, cannabis, and sedatives were used by 74.7%, 74.1%, and 50.9% of participants, respectively. However, these findings differed from those of the study conducted by Andreecia M et al.^25^, in which the most commonly abused substance (barring tobacco products) with opioids was cannabis followed by alcohol. The results from the study by Yasir et al.^21^ reported lifetime use of cannabis, alcohol, and sedatives-hypnotics in 50.33%, 21.33%, and 18.33% of participants, respectively. This difference in the prevalence of use of these substances between our study and the study by Yasir et al. could be because the current study was based exclusively on opioid injectors.

The average age of initiation of injection drug use among our study participants was 21.77 ± 5.65 years. In a Delhi-based study by Gregory Armstrong et al. in 2013^27^, the average age of initiation was lower (15.8 years). This age variation can be explained by the difference in the study population that was targeted by the researchers. Their study was community-based and targeted the hidden group with high injecting behaviour as opposed to our study. Peer pressure was the main reason for injecting behaviour among the majority of our participants, followed by curiosity and fun. This is in agreement with the findings of the study conducted by Samina Farhat et al. in 2015^20^. The pressure to conform (to do what others are doing) can be powerful and hard to resist. A person might feel pressure to do something just because others are doing it. Peer pressure can influence a person to do something relatively harmless or something that has more serious consequences. The negative influence of the peer group is more connected to involvement in risk behaviours^28^. Daily heroin use in the last 3 months was reported by 94.2% of the participants. In the study by Silvia Tortajada et al. in 2012^6^, the majority of the study participants used injection drugs one or two days a week, and only 16.2% used them every day. Nearly one-third of the participants had a family history of substance use. Another important finding of the current study was friends as the major path of referral. Treating one injection drug user can be the motivation for seeking treatment among other PWID, creating a positive cycle.

In our study, psychiatric comorbidities were found in 88.1% of the participants. This is similar to findings in other studies conducted in other parts of India, such as those by Farooqui et al. and Mohanty et al.^29,30^, in which 98.6% and 77.5% of participants had psychiatric comorbidities, respectively. This difference in the prevalence can be explained by the difference in the measurement tools used in these studies. Studies from the West have reported a lower prevalence of psychiatric morbidities, 52.7% by Angelo Giovanni M. et al. in 2011^10^ and 43% by Silvia Tortajada et al. in 2012^6^. Approximately two-thirds of our study participants had more than one psychiatric comorbidity. However, this is slightly higher than the number in the study conducted by Farooqui et al. in 2022^29^. This high burden of psychiatric comorbidities requires rigorous assessment of PWID for multiple disorders other than substance use disorders.

Anxiety disorders comprising panic disorder, agoraphobia, social anxiety disorder, and generalized anxiety disorder were found in most of the participants (66.5%). A slightly higher prevalence (71%) of anxiety disorders was also found in a study by Gregory Armstrong et al.^22^. Other studies had a significantly low prevalence of anxiety disorders^6,25^. The higher prevalence of anxiety disorders can be explained by the unrest in Kashmir and the COVID-19 pandemic during the study period.

Nearly one-third (39.3%) of the participants had an antisocial personality disorder. This finding was consistent (44%) with those of the study by Robert K. Brooner et al. in 1993^31^. In a study by S. Darke et al.^32^, community-based methadone patients, prison inmates enrolled in prison methadone programs and prison inmates with no history of heroin use were interviewed to obtain a diagnosis of antisocial personality disorder (ASPD) and psychopathy. They reported that 44% of community-based methadone patients had ASPD, which is similar to the findings of our study^32^. Furthermore, any history of injecting drugs increases the odds of being diagnosed with an antisocial personality disorder by a factor of 21.01^33^. ASPD poses a major challenge to health care providers, as the outcome remains poor.

Major depressive disorder was found in 17.7% of our study population. A similar prevalence of depressive disorder was found in the studies by Trouiller P et al.^7^ (21.3%), Margoob MA et al.^34^, and Mary E. Mackesy-Amiti et al. (25%)^7,23^. Only 3% of the participants in our study had psychotic disorders. PTSD was found in 8.5% of our study participants, which was in line with the findings by Margoob MA et al.^34^. These findings could be attributed to local political unrest that causes trauma and discontent.

In our study, suicidality (excluding low intent) was experienced by 29.5% of participants. The study by Iskandar et al.^5^ showed a prevalence of suicidal ideation and suicide attempts ranging from 50 to 93% and 43–87%, respectively. There have been substantial variations in the prevalence of suicidality between studies. Suicidality has been difficult to compare owing to different risk assessment methods employed by different researchers^5^ and the inclusion of low intent in calculating the prevalence.

In the bivariate analysis, co-occurring substance use of codeine, oral tramadol, and tapentadol and the use of multiple drugs over time by PWID showed a significant relationship with the development of psychiatric comorbidities. Surprisingly, using a combination of opioids at a single point in time protected against psychiatric conditions in people who inject drugs. This finding of polysubstance use and an increase in psychiatric comorbidities in PWID is in line with those in the studies by Fischer et al.^35^ and Mackesy-Amiti et al.^23^, in which polysubstance use was associated with psychiatric comorbidities.

Cannabis use was significantly associated with major depressive disorder. This is in agreement with the studies by Daniel Feingold et al. and John Horwood et al.^36,37^. There is a potential genetic correlation contributing to the comorbidity of cannabis dependence and major depression. Serotonin (5-HT) may mediate such an association, and there is also evidence for specific risk alleles for cannabis addiction^36^. In our study, cocaine, amphetamine, and inhalant use were positively associated with obsessive–compulsive disorder. Daily heroin intake was negatively associated with psychotic disorders. These findings are similar to those of the study by Trouiller P et al. (2020), in which daily heroin use was negatively associated with major depressive disorder and psychotic disorders in PWID^7^. The hypothesis of an antipsychotic or modulatory effect of opioids on dopamine or on the neurotransmitter system that controls mood has been suggested^38–41^. All substances except amphetamine and daily heroin intake were found to be significantly associated with anxiety disorders.

In the binary logistic regression, younger age was protective against psychiatric comorbidities. This can be explained by the cumulative effect of substances over time on the development of psychiatric comorbidities. This also calls for the need for early intervention among people who inject drugs to prevent the development of psychiatric disorders. Having never been married was also associated with more psychiatric comorbidities. The hypothesis for this finding can be ascribed to a lack of social support. In 1999, Rebecca S. Brienza et al. found that opioid users without a current partner were more likely to be depressed than those with a partner^42^.

### Strengths and limitations

This is one of the first studies from the Kashmir Valley to focus on identifying psychiatric comorbidities in the special group of people who inject drugs. This study used nationally and internationally approved tools for data collection. This study also identified various demographic characteristics and the use of concurrent substances in the development of psychiatric comorbidities among opioid injectors and included patients younger than 18 not previously included in any study. In addition, the prevalence of single and multiple comorbidities not routinely reported in earlier studies was calculated in this study.

This study also has some limitations. This was a single-centre study with a cross-sectional design. This study did not analyse dependence on substances other than opioids. Furthermore, this study included PWID seeking medical help; hence, the results can be extrapolated to this group only.

## Conclusions

This study concluded that in addition to injecting opioids, a substantial number of PWID used multiple substances over time. A higher percentage of multiple psychiatric comorbidities was prevalent among PWID who used opioids. Anxiety disorders (panic disorder, social anxiety disorder, agoraphobia and generalized anxiety disorder) were the most frequent psychiatric comorbidities, followed by antisocial personality disorder. The use of other opioids, such as codeine, tramadol, tapentadol and multiple opioids, over time was associated with a higher frequency of psychiatric comorbidities. Furthermore, the concurrent use of other substances, such as tobacco, cannabis, cocaine, inhalants, sedatives and alcoholic beverages, significantly increased the odds of psychiatric comorbidities. Younger age and the use of a combination of opioids were negatively associated with psychiatric comorbidities. Future research should cover all PWID and should also focus on medical comorbidities and the quality of life of PWID with psychiatric comorbidities. The results of this study suggest that physicians treating PWID should be mindful of psychiatric comorbidities and risk factors associated with such conditions during patient care.

### Recommendations

This study recommends routine screening of opioid injectors for psychiatric comorbidities. In general, physicians who treat PWID in deaddiction centres should refer PWID for the identification of psychiatric comorbidities to relevant specialists. The treating physician should especially focus on patients with a history of prolonged injection drug use, older patients, those using multiple drugs, and patients who have never been married for referral. Policy-makers should include the mandatory screening of PWID for psychiatric comorbidities in deaddiction programs.

## Methods

This study was conducted at the Deaddiction Centre and Addiction Treatment Facility of one of the medical colleges in the summer capital of Jammu and Kashmir, India. This was a single-centre, hospital-based, cross-sectional study that was carried out from March 2021 to August 2022. PWID with a history of injection substance use at least once in the last 3 months^25^ and who were seeking treatment were recruited in this study. People of all age groups and both sexes were included in this study. PWID were identified by urine drug screening by card tests and injection marks. Terminally ill patients, those suffering from life-threatening illnesses and pregnant females were excluded from this study.

### Sample size and sampling procedure

Online Raosoft sample size calculation software was used to determine the sample size^43^. The sample size for this research was based on the prevalence from a previous study that reported that 67% of PWID have psychiatric comorbidities^29^. At a 95% confidence interval and a 5% allowable error, the required sample size was 277.

The study participants were recruited using the consecutive sampling method. The researcher began by inviting a single participant, obtaining informed consent, collecting data, and then moving on to the next participant. If the next participant declined to participate, the researcher extended an invitation to a different participant who arrived to seek treatment.

### Data collection

The sociodemographic profiles of the participants were recorded on the data collection sheet of the approved national survey on patterns and extent of substance use^2^. A semi-structured proforma covered questions on paths of referral, age of initiation of opioid injecting behaviour, reasons for injecting opioids and family history of substance use of the participants. In addition, WHO-ASSIST was used to assess the frequency of opioid injections, previous attempts to reduce opioid use, and the presence of co-occurring substance use^44^. The Mini International Neuropsychiatric Interview-7 (*MINI-7*)^45^ was used to ensure uniformity in the diagnosis of psychiatric disorders. The *MINI-7* is a short, structured diagnostic interview developed for evaluating the most common psychiatric disorders in clinical and research settings. The various disorders included are depressive disorders, bipolar affective disorders, suicidality, anxiety disorders, obsessive–compulsive disorders, psychotic disorders, eating disorders, posttraumatic stress disorder, and antisocial personality disorder. It takes approximately 15 min to administer. The *MINI-7* is an updated version of the *MINI* corresponding to the DSM-5. It is the most widely used tool by mental health professionals and health organizations in more than 100 countries. To assess comorbid psychiatric disorders, the *MINI- 7.02* was administered to the participants. It should be noted that we excluded suicidality while calculating the total prevalence of psychiatric comorbidities. Participants diagnosed with a mental health disorder were offered psychiatric follow-up in the psychiatric outpatient department (OPD).

The researcher received necessary training on face-to-face interviews from the Department of Psychiatry. All potential participants were approached to participate. The purpose of this study, procedures involved and the voluntary nature of participation were explained to the patients. The confidentiality of the data was assured.

### Statistical analysis

The Statistical Package for the Social Sciences (SPSS, version 21, SPSS, Chicago, IL, U.S.A.) was used for data analysis. The data were coded and double checked at entry. The results are presented as frequencies and percentages for categorical variables and means with standard deviations for continuous variables. Bivariate analysis was carried out using Fisher’s exact test, the chi square test with or without Yates correction and odds ratios with 95% confidence intervals to assess the relationship between sociodemographic characteristics and concurrent use of other substances and major psychiatric comorbidities. Logistic regression was carried out using the ENTER method to identify the variables predicting psychiatric comorbidities. The adjusted odds ratio with 95% CI was calculated and is presented in the relevant tables. A *P* value of < 0.05 was considered significant for this study.

### Ethics statement

The institutional Ethical Committee of SKIMS Medical College Srinagar (IEC SKIMS Medical College) approved the research protocol (protocol no: IEC/43/2021 dated 26th March 2021).

### Informed consent

Informed consent was obtained from all participants and/or their parents/guardians before the interviews. Research was performed in accordance with local guidelines/regulations and in accordance with the Declaration of Helsinki. Participation was voluntary, and the data collected were anonymous.

## Data Availability

The datasets used and/or analysed during the current study are available from the corresponding author upon reasonable request.
